# Multidisciplinary management of type 2 inflammation diseases using a screening tool

**DOI:** 10.3389/falgy.2024.1427279

**Published:** 2024-07-18

**Authors:** Oscar Palomares, Carolina Cisneros, Francisco Javier Ortiz de Frutos, José Miguel Villacampa, Ignacio Dávila

**Affiliations:** ^1^ Department of Biochemistry and Molecular Biology, School of Chemistry, Complutense University of Madrid, Madrid, Spain; ^2^ Pneumology Department, La Princesa Hospital, Madrid, Spain; ^3^ Dermatology Department, University Hospital 12 de Octubre, Madrid, Spain; ^4^ ENT Department, Fundación Jiménez Díaz University Hospital, Madrid, Spain; ^5^ Allergy Service, University Hospital of Salamanca, Salamanca, Spain

**Keywords:** T2 inflammation, management, multidisciplinary, screening, questionnaire

## Abstract

Dysregulation of type 2 (T2) immune response leads to an aberrant inflammatory reaction that constitutes the pathophysiological basis of diseases involving various organs. For this reason, several disorders can coexist in a single patient; however, as different specialists often treat these pathologies, T2 dysregulation, particularly when mild, is not always the first diagnostic suspicion. A breakdown in interdisciplinary communication or the lack of adequate tools to detect these entities can delay diagnosis, and this, together with a lack of coordination, can lead to suboptimal care. In this context, a multidisciplinary group of specialists in pneumology, immunology, allergology, dermatology and otorhinolaryngology compiled a list of the cardinal symptoms reported by patients presenting with T2 inflammation-related diseases: asthma, chronic rhinosinusitis, allergic rhinitis, allergic conjunctivitis, IgE-mediated food allergy, atopic dermatitis, eosinophilic oesophagitis, and NSAID-exacerbated respiratory disease (NERD). Using this information, we propose a simple, patient-friendly questionnaire that can be administered at any level of care to initially screen patients for suspected coexisting T2 diseases and referral to the appropriate specialist.

## 1 Introduction

The immune system deploys type 2 (T2) immune reactions as a defence mechanism (1). Dysregulation of this response, however, leads to pathological inflammation and has been implicated in the pathophysiology of various diseases (2). All these diseases share a common endotype, which explains, at least in part, why they frequently coexist and present similar pathophysiological features.

Although advances in understanding the mechanisms underlying T2 inflammation have led to the development of targeted therapeutic strategies, knowledge gaps and unmet needs persist (3). Among the latter, patients themselves have drawn attention to the need for interdisciplinary collaboration in treating T2-mediated diseases (4). Given the frequent coexistence of T2-mediated inflammatory diseases and the fact that mild forms can be overlooked, it is essential for specialists to have access to resources that will promote interdisciplinary communication. Therefore, this perspective article aims to review the main clinical signs and symptoms of T2-mediated inflammatory diseases and to propose a short questionnaire that may aid diagnosis and improve the early detection of coexisting pathologies by referring the patient to the appropriate specialist.

## 2 Cardinal signs and symptoms of pathologies presenting with T2 inflammation

We searched MEDLINE (PubMed) and Scopus for articles describing the characteristic symptoms of the eight most prevalent diseases associated with T2 inflammation [asthma, chronic rhinosinusitis, allergic rhinitis, allergic conjunctivitis, IgE-mediated food allergy, atopic dermatitis, eosinophilic oesophagitis, and NSAID-exacerbated respiratory disease (NERD)]. We used general search terms (“type 2 inflammation”, “type 2 response”, “type 2 immunity”, “disease”, “clinical symptoms”, “clinical presentation”, “aetiology”, “consensus”, “management”, “guidelines”) combined with the names of each pathology and filtered for publications in English or Spanish from the last 5 years. Several meetings were held to discuss the articles retrieved from the search and to pool our clinical experience in these diseases.

### 2.1 Asthma

Asthma has an estimated prevalence of 5%–10% (5). It is over- and underdiagnosed, so there is considerable room for improvement in diagnosing this entity (6). Characteristic symptoms include shortness of breath, cough (usually dry and persistent), wheezing, and chest tightness (7). Although persistent dry cough is non-specific, it is one of the cardinal signs of asthma and should raise diagnostic suspicion, particularly in children, even in the absence of other symptoms.

### 2.2 Chronic rhinosinusitis

T2 inflammation is usually associated with patients with nasal polyposis (CRSwNP) (8), although it has been described as very common in patients with chronic rhinosinusitis without nasal polyps (CRSsNP) from Western populations (9). Diagnosis of chronic rhinosinusitis in adults is based on symptoms and endoscopic or computed tomography (CT) findings. Patients must present two or more symptoms, one of which must be nasal obstruction or rhinorrhoea, with or without hyposmia/anosmia or facial pain/pressure, lasting more than 12 weeks (10). In CRSwNP patients, the most frequent signs and symptoms are nasal obstruction, runny nose, and/or postnasal drip, together with hyposmia/anosmia. Nevertheless, we could say that nasal obstruction and loss of smell are the cardinal symptoms of CRSwNP.

### 2.3 Allergic rhinitis

This type of rhinitis can be intermittent or persistent, as stated by the Allergic Rhinitis and its Impact on Asthma (ARIA) guidelines (11). In many countries, most patients with allergic rhinitis seen in routine practice present symptoms year-round, although allergic rhinitis is strongly suspected when symptoms recur during periods of exposure and disappear otherwise. Another factor to consider is symptom intensity rated according to the criteria established in the ARIA guidelines (12). Allergic rhinitis manifests clinically with nasal congestion, rhinorrhoea, nasal and/or pharyngeal itching, and sneezing paroxysms, and is often accompanied by conjunctival symptoms due to its frequent association with allergic conjunctivitis (13). The most common clinical symptoms reported by patients are sneezing and nasal itching.

### 2.4 Allergic conjunctivitis

Recent data suggest that the prevalence of allergic conjunctivitis is steadily increasing (14), and it is often associated with allergic rhinitis. Despite its impact on quality of life, diagnostic delay, underdiagnosis, and self-diagnosis are common, and many patients self-medicate (15). Although patients with allergic conjunctivitis may occasionally present other ocular symptoms, such as eyelid oedema or photophobia, the cardinal signs are pruritus, tearing, and conjunctival hyperaemia, which may be periodic (seasonal allergic conjunctivitis) or chronic (perennial allergic conjunctivitis) (16).

### 2.5 IgE-mediated food allergy

IgE-mediated food allergy is usually acute, and symptoms usually develop within 1 h of ingestion (17). Symptoms can involve virtually any organ or system (skin, respiratory, gastrointestinal, cardiovascular, neurological) and vary in severity. Acute urticaria or rash, and gastrointestinal symptoms, are the most frequent manifestations of food allergy, although it can also include upper and lower airway issues, angioedema and even hypotension. A common type of food allergy is oral allergy syndrome (OAS), the main symptoms being pruritus of the oral mucosa and tongue that develop rapidly after ingestion of the triggering allergen in the absence of systemic symptoms.

### 2.6 Atopic dermatitis (AD)

The coexistence of AD with other allergic pathologies is so widespread that allergic manifestations are even considered in the diagnostic criteria included in some clinical guidelines (18). AD is usually easily diagnosed in children, although more severe forms of the disease and adult AD are more challenging to diagnose (19). AD can present with various types of lesions in different locations. For example, patients may present erythematous and oedematous smooth plaques, exudates with or without skin crusts, desquamation, lichenification, or erythematous and excoriated papules, all of which are highly pruritic. Chronic erythematous lesions or flare-ups and intense pruritus are the most characteristic signs reported within this variety. Adult patients typically have lesions on the head, neck, hands, and feet.

### 2.7 Eosinophilic oesophagitis

Diagnosis and follow-up of eosinophilic oesophagitis are based on symptoms and histology (20). Although symptoms are chronic in some patients, in others, they are intermittent, and they will remain asymptomatic between exacerbations (21). Signs and symptoms in children and adolescents may vary with age indeed, younger children will present with abdominal pain, feeding problems or refusal to eat, and vomiting. However, cardinal symptoms suggestive of eosinophilic oesophagitis in adults are dysphagia and food impaction.

### 2.8 NSAID-exacerbated respiratory disease (NERD)

Patients with NERD present the triad of asthma, eosinophilic CRSwNP, and acute respiratory symptoms after taking cyclooxygenase-1 (COX-1) selective NSAIDs (22). These patients often have severe asthma and tend to have recurrent severe nasal polyposis and lower baseline lung function than patients with NSAID-tolerant asthma (23). The syndrome generally onsets between the ages of 20 and 40 years and manifests as upper and lower airway symptoms attributable to inflammation caused by CRSwNP and asthma, respectively. However, clinicians should suspect NSAID hypersensitivity when the patient presents respiratory symptoms (nasal congestion, runny nose, wheezing, bronchospasm), often severe, after ingesting an NSAID.

## 3 A screening tool for suspected T2 inflammation-related pathologies

Despite broad evidence that T2 inflammation often underlies many different diseases, factors such as poor interdisciplinary coordination, excessive focus on severe manifestations, and the absence of appropriate screening tools frequently delay treatment and diagnosis, resulting in suboptimal quality of care. In a recent European study, patients themselves highlighted a lack of a multidisciplinary approach as the major unmet need in these diseases (4). Few studies have analyzed the interdisciplinary approach to these pathologies, and there are no updated clinical guidelines (3).

We pooled our expertise in various specialist fields to develop the screening tool presented in this paper, in order to address the unmet need for rapid identification by primary care doctors and specialists of the different concomitant T2 inflammatory diseases. A diagnostic instrument used at all levels of care to promptly detect T2 inflammation as the underlying cause in certain patients could promote interdisciplinary collaboration and timely referral of these patients. The tool presented here is a screening questionnaire that detects the cardinal signs and symptoms of various pathologies that are likely to coexist (Table 1, Supplementary Table S1). We performed an extensive literature search for evidence relating to the characteristic clinical signs reported by patients in each case and held a series of meetings to compare these results with our clinical experience and to draw up the questionnaire. Instead of medical terminology, we drafted the questions using the language patients usually use when describing their symptoms. This study follows the approach employed in a recent checklist for the multidisciplinary approach to the united airway, a concept that encompasses asthma and chronic rhinitis/rhinosinusitis (24). Unlike our study, the main aim, in that case, was to standardize the criteria for data collection to allow the data to be used in clinical research. Our questionnaire is quick and easy to administer within the time limits of a typical consultation and covers all the main pathologies known to derive from T2 inflammation. It will be of benefit in all healthcare contexts, although it could play a particularly important role in primary care, enabling clinicians to focus on T2 inflammation from the onset of symptoms by screening for coexisting pathologies.

**Table 1:** Questionnaire for the initial screening of T2 inflammation-related pathologies.

Question^a^	Answer (yes/no)	Diagnostic suspicion^b,c^
1. Do you have persistent or long-term dry cough?		Asthma
2. Is it difficult for you to breathe even when you are not making a physical effort?		
3. Do you usually have a stuffed-up nose?		Chronic rhinosinusitis with nasal polyps
4. Do you feel that you have completely or partially lost your sense of smell?		
5. Do you sneeze frequently or during specific periods of the year?		Allergic rhinitis
6. Does your nose and/or palate itch regularly or during specific periods of the year?		
7. Do your eyes itch frequently or during specific periods of the year?		Allergic conjunctivitis
8. Are your eyes red and/or watery regularly or during specific periods of the year?		
9. Have you noticed that your lips, mouth, or throat itch after eating certain foods?		IgE-mediated food allergy
10. Have you suddenly felt shortness of breath, dizziness, itching or skin lesions all over your body, or swelling on your face after eating certain foods?		
11. Do you often develop a very itchy rash or red patches on your skin?		Atopic dermatitis
12. Do you notice that your skin is thicker or rougher in areas where you scratch frequently?		
13. Do you sometimes have trouble swallowing food or even feel nauseous and regurgitate recently swallowed food?		Eosinophilic oesophagitis
14. When you eat, do you sometimes notice a kind of knot or discomfort in your chest or in the pit of your stomach that prevents you from continuing to swallow?		
15. Have you noticed that your respiratory symptoms develop or worsen (congestion, shortness of breath, runny nose, etc.) shortly (1–3 h) after taking an aspirin, ibuprofen, or other anti-inflammatory?		NERD

NERD, NSAID-exacerbated respiratory disease.

^a^ The questions are formulated using a language that can be easily understood by any person or patient with no medical training.

^b^ Referral should be considered if at least one answer is affirmative.

^c^ In the patient-facing version of the questionnaire, this column will remain empty to mitigate potential biases in their responses.

Even though this questionnaire is based on an extensive literature review and has been constructed by expert consensus, its future validation with patients using the appropriate methodology is under development. For that purpose, the language and construction of items will be explored, and synonyms and variations for the same concept will be assessed. Subsequently, all questionnaire variants will be tested following the Delphi method with physicians from each specialty and a sample of patients recruited from patient advocacy groups. The resulting final version of this tool will be used in a validation study performed in clinical practice. Therefore, the questionnaire will be administered to patients from the pilot centres diagnosed with a T2 inflammation pathology, and variables such as rate of cross-referral or time to diagnosis after referral to another specialty, among others, will be prospectively recorded and analysed. Once the questionnaire has been validated, possible derived actions would involve evaluating real-world models of tool application, such as in primary care or other specialties, or implementing the tool prior to any referral.

## 4 Conclusion

Several different T2 inflammation-related diseases often coexist in the same patient. However, the tendency among clinicians to focus on the most serious pathology and overlook other less severe diseases leads to diagnostic delays. In this perspective study, we reviewed the cardinal and differential symptoms of eight pathologies in which T2 inflammation is the underlying cause and propose a simple, quick, patient-friendly questionnaire that can be administered at any level of care. We believe this tool will improve the existing approach to these pathologies by facilitating early suspicion of T2 involvement and allowing clinicians to treat them as a group.

## Data Availability

The original contributions presented in the study are included in the article/Supplementary Material, further inquiries can be directed to the corresponding author.
