# Supplementary material for: Multidisciplinary management of type 2 inflammation diseases using a screening tool
Source: Front Allergy. 2024 Jul 18;5:1427279. doi: 10.3389/falgy.2024.1427279 (PMC11291315; doi:10.3389/falgy.2024.1427279)
Supplement: Supplementary file 1 [file Table1.docx]

**Table S1**. Cuestionario para el cribado inicial de patologías asociadas a inflamación T2 (versión en español)

| **Pregunta*** | **Respuesta (sí/no)** | **Sospecha diagnóstica^†^**^¥^ |
| --- | --- | --- |
| 1. ¿Tiene tos seca de manera continua o durante largos periodos de tiempo? |  | Asma |
| 1. ¿Le cuesta respirar incluso sin haber hecho esfuerzo físico? |  |  |
| 1. ¿Tiene la nariz congestionada de forma habitual? |  | Rinosinusitis crónica con pólipos nasales |
| 1. ¿Siente que ha perdido el olfato o huele peor? |  |  |
| 1. ¿Estornuda con mucha frecuencia de forma habitual o durante algunos periodos del año? |  | Rinitis alérgica |
| 1. ¿Le pica la nariz y/o el paladar de forma habitual o durante algunos periodos del año? |  |  |
| 1. ¿Le pican los ojos de forma habitual o durante algunos periodos del año? |  | Conjuntivitis alérgica |
| 1. ¿Nota los ojos enrojecidos y/o llorosos de forma habitual o durante algunos periodos del año? |  |  |
| 1. Después de comer algún alimento, ¿ha notado picor en los labios, la boca, o la garganta? |  | Alergia alimentaria mediada por IgE |
| 1. Después de comer algún alimento, ¿ha sentido de forma repentina dificultad para respirar, mareo, picor por el cuerpo o lesiones en la piel, o hinchazón en la cara? |  |  |
| 1. ¿Tiene sarpullidos o manchas rojas en la piel que le producen mucho picor con frecuencia? |  | Dermatitis atópica |
| 1. ¿Nota la piel más gruesa o rasposa en las zonas donde se rasca frecuentemente? |  |  |
| 1. ¿En ocasiones tiene problema para tragar los alimentos o incluso presenta náuseas y regurgitaciones? |  | Esofagitis eosinofílica |
| 1. Al ingerir alimento, ¿en ocasiones nota una especie de nudo o molestia en el pecho/boca del estómago que no le permite seguir tragando? |  |  |
| 1. ¿Ha presentado desarrollo o empeoramiento de síntomas respiratorios (congestión, dificultad para respirar, goteo por la nariz…) al poco tiempo (1-3 horas) de tomarse una aspirina, ibuprofeno u otro antiinflamatorio? |  | EREA |

^*^ Las preguntas están formuladas con un lenguaje adaptado a la fácil comprensión de cualquier persona o paciente sin formación médica.

**^†^** La derivación debería considerarse en caso de al menos una respuesta afirmativa.

^¥^ En la versión del cuestionario dirigida al paciente, esta columna permanecerá vacía para mitigar posibles sesgos en sus respuestas.

EREA, enfermedad respiratoria exacerbada por AINE
